# Current Understanding and New Advances in the Surgical Management of Reparable Rotator Cuff Tears: A Scoping Review

**DOI:** 10.3390/jcm12051713

**Published:** 2023-02-21

**Authors:** Franziska Eckers, Stefan Loske, Eugene T. Ek, Andreas M. Müller

**Affiliations:** 1 Orthopädie und Traumatologie, Universitätsspital Basel, 4031 Basel, Switzerland; 2 Melbourne Orthopaedic Group, Melbourne, VIC 3181, Australia; 3 Department of Surgery, Monash University, Melbourne, VIC 3168, Australia

**Keywords:** rotator cuff, shoulder, advances, pathology, arthroscopy, MR arthrography, double row, augmentation

## Abstract

Rotator cuff (RC) tears are among the most common musculoskeletal disorders and can be associated with pain, weakness, and shoulder dysfunction. In recent years, there have been significant advances with regard to the understanding of rotator cuff disease and its management. With technological improvements and advanced diagnostic modalities, there has been much progress as to improved understanding of the pathology. Similarly, with advanced implant designs and instrumentation, operative techniques have evolved. Furthermore, refinements in postoperative rehabilitation protocols have improved patient outcomes. In this scoping review, we aim to provide an overview of the current knowledge on the treatment of rotator cuff disorders and to highlight recent advances in its management.

## 1. Introduction

Rotator cuff disorders are among the most common musculoskeletal conditions and can be associated with severe pain, weakness and shoulder dysfunction [1,2]. Their prevalence depends not only on age, but also on factors, such as patient dexterity, history of trauma, and occupation [3,4]. It ranges from 10% in the young adult population to over 60% in the 80+ generation [3,4,5]. The degree of symptoms varies substantially, with the majority of affected individuals being asymptomatic [3]. However, those patients who do develop symptoms may sometimes experience disabling pain and loss of function that may inhibit them in their daily lives, recreational pursuits, and occupation. The causes of these symptoms are variable and are not conclusively understood [6].

Treatment options include nonoperative measures as well as a wide variety of surgical treatments. In addition to the severity and duration of symptoms, decision-making considers a variety of other factors, such as tear etiology and morphology and the expected tissue quality of the muscle–tendon unit, as well as the patient’s overall profile, expectations, and ability for the patient to undergo adequate rehabilitation.

In recent years, research has contributed notably to the existing body of knowledge on rotator cuff disease and management. Moreover, technological improvements have significantly expanded the range of diagnostic and operative techniques, as well as the available instruments and implants. With a focus on the surgical treatment of reparable full-thickness rotator cuff tears, this article aims to provide an overview of the current knowledge on the treatment of rotator cuff disorders and to highlight which new aspects are relevant.

## 2. Pathoanatomy and Pathogenesis

The pathogenesis of rotator cuff tears is multifactorial and is still not clearly understood [7,8]. Far more common than traumatic are degenerative tears or traumatic on degenerative tears, in which both extrinsic mechanical factors and intrinsic factors, in the tendon itself, play a role [9]. Neer, Bigliani, and Rockwood studied the architecture of the acromion in the 1970s to 1990s and found “impingement” of the rotator cuff under the coracoacromial arch [10,11,12] to be primarily responsible for the development of rotator cuff lesions. This theory has steered surgeons for many years to perform stand-alone or adjunctive acromioplasties. However, the described pathomechanism has since been challenged and a number of studies have invalidated the efficacy of acromioplasty in the treatment of subacromial pathologies [13], the most prominent being that of Beard et al. published in *The Lancet* in 2018 [14]. At present, from a mechanical viewpoint, tensile overload of the supraspinatus tendon, rather than a spur digging into the tendon, is believed to be primarily responsible for tendon failure, especially in the presence of certain omometric features [15]. As such, it has been recognized that the lateral extension of the acromion, which can be objectified on the standard anteroposterior (AP) radiograph by measuring the acromion index (AI) [16] or the critical shoulder angle (CSA) [8,17], is associated with increased incidence of rotator cuff tears [18]. The reason is a decrease in compressive and an increase in shear forces around the glenohumeral joint, leading to joint instability and necessitating more supraspinatus force, resulting in more load on the tendon [15]. Moreover, recent results suggest that a greater sagittal extension and downward slope of the acromion, as well as the length–width ratio of the acromion and the offset of its medial border with regard to the glenoid plane, may also be relevant [19,20]. This research is ongoing. 

At least equally important to the pathogenesis of degenerative rotator cuff tears are intrinsic factors. The tendon tissue itself undergoes life-time-dependent changes leading to mucoid degeneration as well as the incorporation of hydroxyapatite microcalcifications [21,22,23,24]. In addition, the tendon attachment zone, the rotator crescent, is hypovascular [25,26]. Repetitive tensile, compressive, and shear stresses lead to tissue overload and a tendon disease continuum with the progression of tendinopathic changes into partial tears and eventually transmural tears [6,27]. Risk factors include smoking, genetic factors, and metabolic diseases [28,29,30,31]. Among the latter, thyroid conditions as well as diabetes mellitus are particularly noteworthy as they affect the tendon tissue directly in an adverse way [30,31,32]. In vitro experiments have shown that thyroid hormones enhance tenocyte growth while counteracting apoptosis in healthy tenocytes [31]. On the other hand, nonenzymatic, oxidative reactions between glucose and collagen in patients with diabetes mellitus seem to negatively influence the biomechanics of tendon tissue [32]. New biological therapeutic approaches, which may be effective here in the future, will be discussed during the course of this review article.

## 3. Imaging

Imaging of the shoulder joint has seen significant advances in the last decade. Different questions require the use of different modalities, which may be used complementarily to each other in some instances. Ultrasound, computed tomography (CT), and magnetic resonance imaging (MRI) are all commonly used modalities for imaging of the rotator cuff. Thus, in those situations where the modality of choice is not available, there are usually alternative options. 

Ultrasound is a very reliable method in the hands of an experienced examiner, at least with respect to the detection of full-thickness tears, but also for the assessment of partial tears or tendinopathy, rotator cuff muscle tropism, pathologies of the long head of biceps tendon, bursal abnormalities, AC joint pathologies, and some other findings, such as cysts (i.e., spinoglenoid) or compression of neural structures (i.e., suprascapular nerve) [33]. It has the added advantage of being a dynamic method of examination and is often used in the early examination of rotator cuff disorders or in the context of repeat examinations both in nonsurgical and surgical patients [34,35]. For therapeutic decision-making, however, surgeons tend to rely on cross-sectional imaging, if available. Thus, in the context of this article, we would like to focus on these techniques. Here, there have been some interesting advances and developments in recent years. 

### 3.1. Magnetic Resonance Imaging (MRI) versus MR Arthrography (MRA)

**Figure 1:**
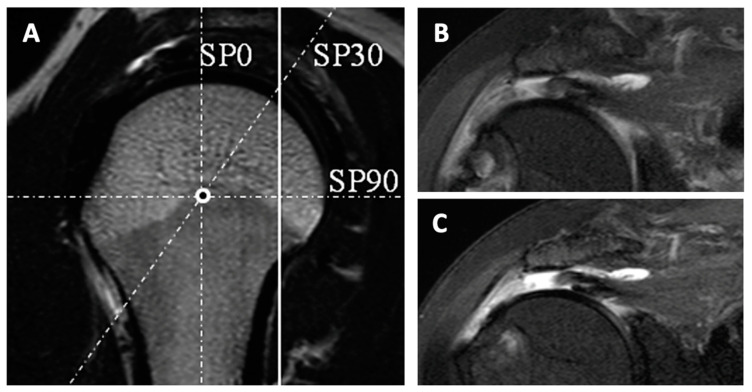
(**A**) Illustrates examples of both conventional oblique coronal MRI slices (continuous line) and radial MRI slices (interrupted lines). The region of interest is the posterosuperior rotator cuff, where the continuous line and the interrupted SP30 line meet. (**B**) In the oblique coronal image, the posterosuperior rotator cuff tear is detectable. (**C**) The radial image allows for better characterization of the retracted and delaminated tear [37]. (Reproduced from with permission from Ref. [37]. Copyright © 2023, published by Elsevier Masson SAS. All rights reserved.)

MRI is most commonly the modality of choice for diagnosing pathology involving the rotator cuff. Routine protocols include sequences in oblique axial, sagittal, and coronal planes with both T1-weighted and fluid-sensitive sequences [36]. They allow for a detailed assessment of the tendon morphology. Pathological features can be detected with a high degree of accuracy. Recently, a technique that can further visualize certain “blind spots” has been introduced; radial sequences, as opposed to The traditional 90° planes, permit even more complete visualization of the footprint and the rotator interval. Several authors [37,38,39] have demonstrated their superiority in detecting and clearly visualizing certain cuff tears, especially partial tears, and tears that are located posterosuperiorly, but also at the level of the rotator interval (Figure 1) [37].

**Figure 2:**
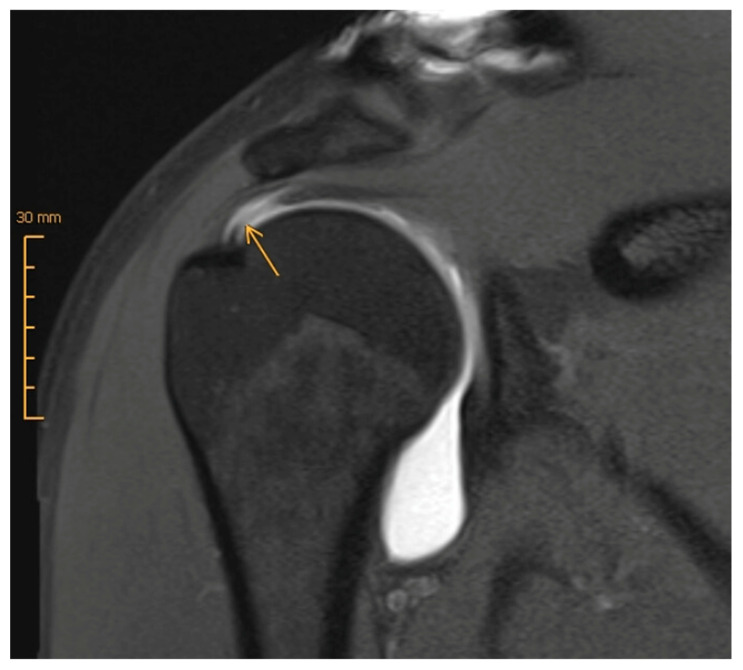
MRA (t1_tse_cor) with partial-thickness articular surface rotator cuff tear (yellow arrow).

**Figure 3:**
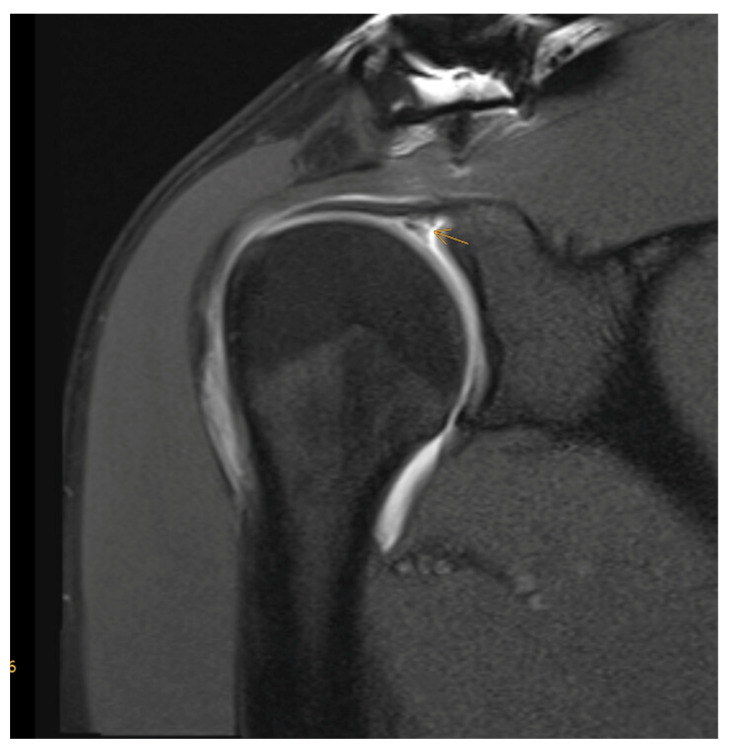
MRA (t1_tse_cor) with SLAP (superior labrum anterior to posterior) lesion (yellow arrow).

What about the use of intra-articular contrast agent? According to a 2015 meta-analysis, MRI and MRA, as well as sonography, are on par for detecting full-thickness tears, with sensitivities and specificities of at least 0.9. However, with respect to the diagnosis of partial tears, MRA is the most sensitive method [34] (Figure 2). Furthermore, recent studies have shown that the use of intra-articular contrast agent clearly enhances the imaging quality and diagnostic accuracy of pathologies of the long biceps tendon, its anchor and its retaining apparatus (Figure 3), which often coexist with rotator cuff injuries [40,41]. Moreover, a 2021 prospective study by Groarke, which included 200 patients undergoing MRI or MRA followed by shoulder arthroscopy, reported a better correlation between preoperative and intraoperative findings for MRA (*p* = 0.002) [40]. The question remains whether these advantages of MRA relative to MRI are pertinent enough to justify on one hand the additional effort and cost that is required, and on the other hand the increased risk and discomfort that is associated with joint infiltration, such as pain, infection, and allergic reaction. In 2009, Magee analyzed a cohort of 150 patients who each received both MRI and MRA. In almost 20%, the treating orthopedic surgeon changed the treatment strategy based on the additional findings detected on the MRA [41]. Therefore, its application in certain circumstances and for specific questions (rotator cuff partial rupture, labral lesion) or as an additional examination in unclear cases can be useful.

### 3.2. Assessment of Muscle Quality

**Figure 4:**
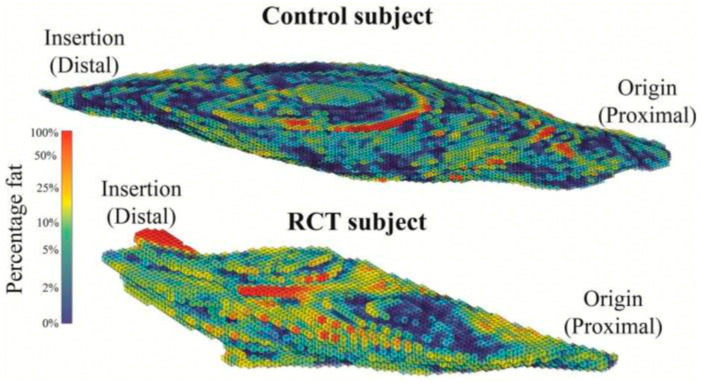
This figure shows the fat percentage of supraspinatus in a control subject (above) and a subject with a rotator cuff tear (RCT) (below) based on a 3D Dixon MRI [48]. (Reproduced from with permission from Ref. [48]. Copyright © 2023, published by Springer Nature. All rights reserved.)

The assessment of muscle quality and fatty muscle infiltration is one of the most important criteria that largely dictates reparability of rotator cuff tears [42,43]. To date, it has been assessed by applying the Goutallier/Fuchs criteria [42,44,45]. Presence and extent of fatty muscle infiltration are subjectively assessed by the examiner on parasagittal CT/MRI and graded between zero and four. However, the interrater reliability for this classification is known to be only satisfactory [46]. Therefore, several new technological advances to objectify and quantitatively analyze the fat content and the fat-to-muscle ratio in the rotator cuff have been recently proposed, including spectroscopic methods and T2 mapping [47,48]. Figure 4 illustrates an example of a method that is based on 3D Dixon MRIs, which separates fat from water, with subsequent segmentation and calculation of fat percentage [48]. This or similar techniques may in the future allow to measure both fat percentage and muscle atrophy and to draw assumptions as to the probability of retearing [36,49]. Other methods have been based on the use of CT scan. So far, all techniques that aim to quantify the fat fraction in rotator cuff muscles have been used in the context of studies or experimentally. Still to date, a gold standard does not yet exist. Therefore, the results of the current research into this field may in the future influence clinical decision-making as to cuff reparability and prognosis. 

**Figure 5:**
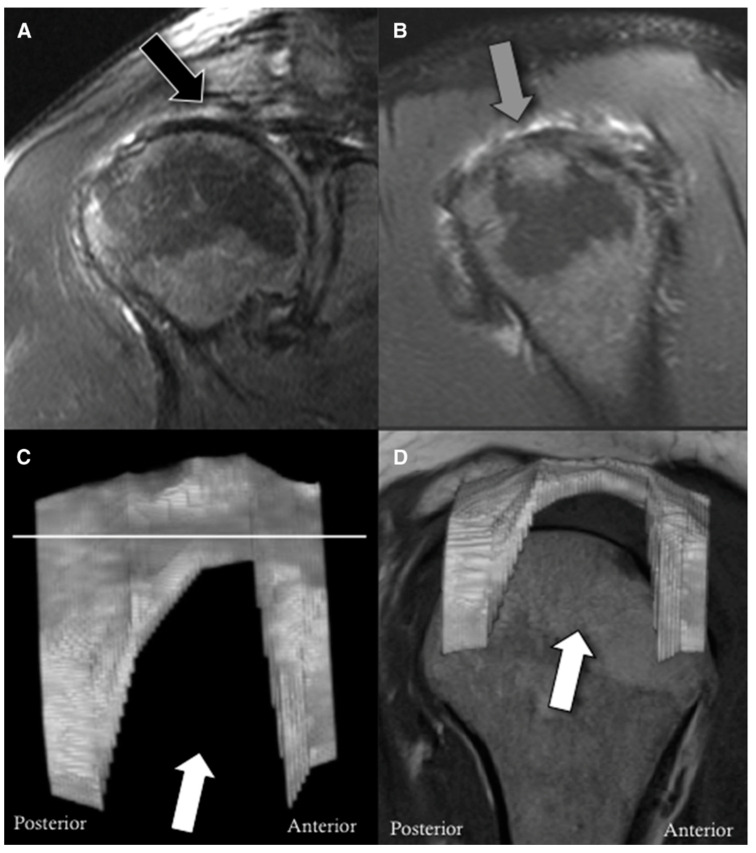
A 50-year-old man with a U-shaped rotator cuff tear. (**A**) Coronal and (**B**) sagittal fat-suppressed T2-weighted images of the right shoulder demonstrate a full-thickness tear of the supraspinatus tendon (black and grey arrow). (**C**,**D**) demonstrate 3D reconstructions of the supraspinatus and anterior infraspinatus tendons including a U-shaped tear (white arrows). The white line in C denotes the joint line [50]. (Reproduced from with permission from Ref. [50]. Copyright © 2023, published by Elsevier. All rights reserved.)

Lastly, in relation to MR imaging of the rotator cuff, as with CT scan, three-dimensional MR reconstructions are now also possible. Hence, as seen in Figure 5, with the aid of postproduction software, 3D reconstructions that may facilitate the visualization of the rupture pattern (i.e., crescent vs. U- vs. L-shape) can be generated. A 2015 study by Gryfpopoulos could confirm that 3D reconstruction of cuff tears does indeed improve the accuracy of the interpretation of the images [50]. This invariably may allow for better preoperative planning and can also help in decision-making as to the need for potential augmentation. Nevertheless, it still remains to be seen if or when this technology will find its way into everyday clinical practice.

## 4. Surgical Repair of Rotator Cuff Tears

Treatment options for rotator cuff tears remain diverse and include a wide spectrum of conservative measures, such as different types of physiotherapy and infiltrative interventions, as well as surgical treatment methods, including rotator cuff debridement and acromioplasty, partial repair, tendon repair with or without augmentation, rotator cuff reconstruction using interpositional techniques or tendon transfers, superior capsular reconstruction, or reverse shoulder arthroplasty [51,52]. Indications often overlap and depend—respecting certain principles—also on the “personality” of both the tear and the patient that is being treated. Besides etiology and morphology of the tear, symptom duration and severity, the presence of degenerative arthritis, the patient’s compliance, and their physical demands all need to be taken into account. More often than not, there are mixed situations, gray areas, and a variety of influencing factors, which sometimes make it difficult to clearly assign patients to the appropriate treatment option. 

Regarding the treatment of reparable full-thickness rotator cuff tears, to date, clear evidence of superiority of surgical repair over conservative treatment is lacking [53,54]. This has been shown, i.e., in a 2021 meta-analysis by Longo et al. [53]. Nonetheless, a 2014 publication by Moosmeyer et al. [54] which presented detailed results of 103 patients who were randomized into a physiotherapy and a primary tendon repair group did report somewhat better outcomes for patients with primary tendon repair at the 5-year follow-up, even if not of clinical importance. This article also indicates that one-third of patients in the physiotherapy group underwent secondary tendon repair due to insufficient treatment success. Moreover, one-third of the remaining 38 conservatively treated lesions relevantly increased in tear size, which was associated with a poorer outcome. A previous meta-analysis including three level I and II studies comparing conservative with operative treatment published by Piper et al. [55] in 2018 also concluded that operative treatment was slightly advantageous when compared to conservative treatment. It was however stated that both forms of treatment reliably improved functional outcome and decreased pain. The follow-up period was only 12 months.

In the following, we shall highlight the principles of surgical treatment of the reparable rotator cuff as well as some new advances with regard to techniques and technologies.

### 4.1. Indications and Timing

Appropriate management of rotator cuff pathology needs to take into consideration both the natural history if left untreated and also the likelihood of healing of the tears if repaired. In 2019, Keener et al. [25] reported on the natural course of 224 asymptomatic shoulders and found that the process of deterioration of degenerative tears is overall slow, but that tears involving the rotator cable were predisposed for rapid progression. Moosmayer described similar dynamics, but also showed that tear size progression is accompanied by increasing fatty muscle infiltration [56]. Studies that looked at tendon healing after surgical therapy identified older patient age (>65 years), greater tear size and retraction, and a higher degree of fatty infiltration and a shorter tendon stump as major risk factors for nonhealing of a reconstructed tendon [42,56,57]. In addition to these aspects concerning the pathogenesis of rotator cuff pathology, the lengthy rehabilitation period that patients must undergo after rotator cuff reconstruction needs to be considered and explained to each surgical candidate. Despite any advances in treatment, the time required to rehabilitate the shoulder after rotator cuff repair remains unchanged, requiring at least 6 months before pain and functional level reach adequate levels [58]. 

Rotator cuff tears may be situated superiorly, posterosuperiorly, anteriorly, or anterosuperiorly. It is known that certain tear patterns cause more severe functional impairment than others. The most commonly used classification with regard to involvement of different tendons/tendon parts in so-called massive rotator cuff tears is that of Collin et al. [59]. It differentiates five types of tears: type A, supraspinatus and superior subscapularis; type B, supraspinatus and entire subscapularis; type C, supraspinatus, superior subscapularis, and infraspinatus; type D, supraspinatus and infraspinatus; and type E, supraspinatus, infraspinatus, and teres minor tears. Collin’s study revealed that the ability to externally and internally rotate is dependent on the amount of involvement of the anterior or posterior rotator cuff. Most importantly, it showed that patients with type B tears were found to be pseuodoparalytic in the majority of cases (80%), and patients with type C and E tears often (45%, 33% respectively), meaning that they were not able to actively forward elevate the arm beyond 90°. It was concluded that the subscapularis is more important than the infraspinatus and teres minor in shoulder elevation. Thus, repair of the subscapularis tendon would be critical for reversing pseudoparalysis.

Each therapeutic decision must be made individually, aiming to achieve pain relief and restoration of function as reliably as possible, while avoiding complications. In younger patients with good healing potential and for tears that may favor rapid progression (large tear, involvement of the rotator cable, incipient fatty infiltration of the musculature), earlier surgical intervention is recommended [55,60]. On the other hand, older patients, patients with small full-thickness or partial lesions, or asymptomatic patients may not require surgery, and a trial of conservative with dedicated physiotherapy, pain management, and potentially a cortisone injection maybe all that is necessary to manage their symptomatology. In these patients, appropriate follow-up should be instituted in the event that their symptoms progress and conservative management fails. In contradistinction, traumatic rotator cuff tears, especially large/massive tears, should be addressed timely. Some authors suggest that they be operated preferably within 3 months of injury, as late reconstruction is associated with comparatively poorer results [61,62]. According to a 2022 review by Van der List [63], on the other hand, early intervention may not be as important as previously believed, at least with respect to retear rate, for which the one-year mark postinjury was found to be the turning point. Notably, the examined clinical outcome scores were still better in the group operated within 3 months, however lacking clinical significance. Table 1 summarizes the prognostic factors that are typically associated with a good outcome after surgical treatment of rotator cuff tears.

### 4.2. Fixation Techniques

As previously mentioned, rotator cuff tears vary in location, shape, and extent. Supra/infraspinatus ruptures may be crescent-, U-, or (reversed) L-shaped in configuration. Subscapularis tears typically occur as “ruptures in continuity” with progressive tendon lengthening and muscle shortening [64].

**Figure 6:**
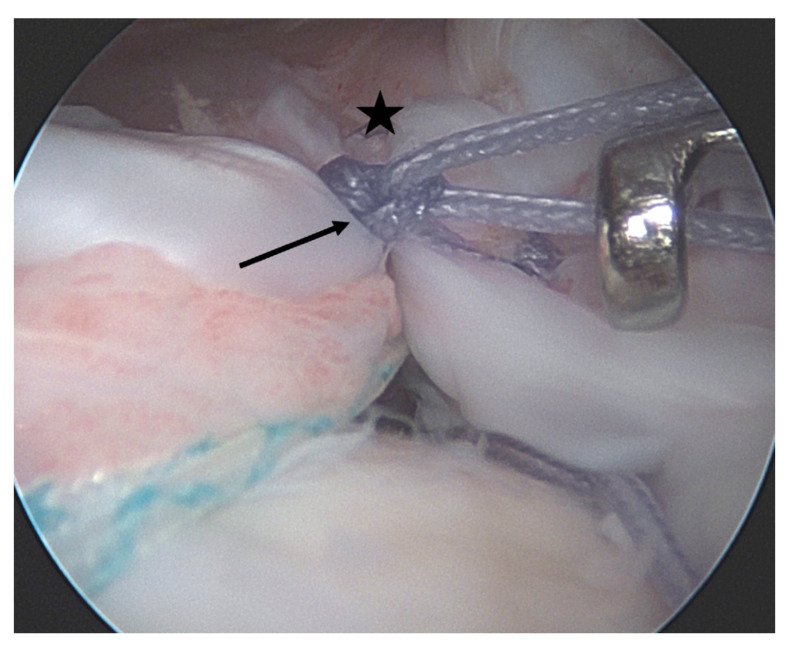
“Margin convergence”. The star indicates the apex of a U-shaped posterosuperior rotator cuff tear. The arrow indicates the stitch used to approximate the anterior and posterior edge of the tear, thus converging the free margin of the tear toward the footprint.

Two specific technical tools that may aid the reconstruction of certain tear types will be briefly discussed here. The first is the concept of “margin convergence” (MC), which was first introduced by Burkart et al. in 1996 [65]. It describes the side-to-side adaptation of a U-shaped rupture’s apex—which lies by definition medial to the glenoid—before reduction to the footprint, thus converting it to a crescent-shaped tear (Figure 6). In doing so, the mediolateral dimension of the defect is reduced while the cross-sectional area of the rotator cuff at the point of pull is increased, consequently lowering the strain on the repair [66,67]. However, retear rates in U-shaped rotator cuff tears treated with this technique may still be high. Kim et al. [68] reported retears in almost half of the observed patients. These were, however, smaller than the original tears and were not associated with poorer clinical outcomes when compared to the fully healed tendons. In a 2020 study with 7.5 years of follow-up, Baumgarten also documented similar clinical results in patients with margin convergence repairs and conventional rotator cuff repairs [69]. The second tool concerns the management of subscapularis tendon tears [70,71,72]. As to treatment indication, data regarding small ruptures (Lafosse I/II) are currently still controversial [72,73], while it is recommended that larger subscapularis tears should be repaired as subscapularis integrity and function are crucial for shoulder kinematics and balance of the glenohumeral joint [71,74,75]. In terms of the operative technique of anterosuperior ruptures, reconstruction of the subscapularis tendon while preserving the “comma sign” helps to understand the anatomy of the tear, on the one hand, and results in a shift of the rotator interval tissue laterally [76], on the other hand. This can also greatly facilitate subsequent reconstruction of the superior rotator cuff [77].

**Figure 7:**
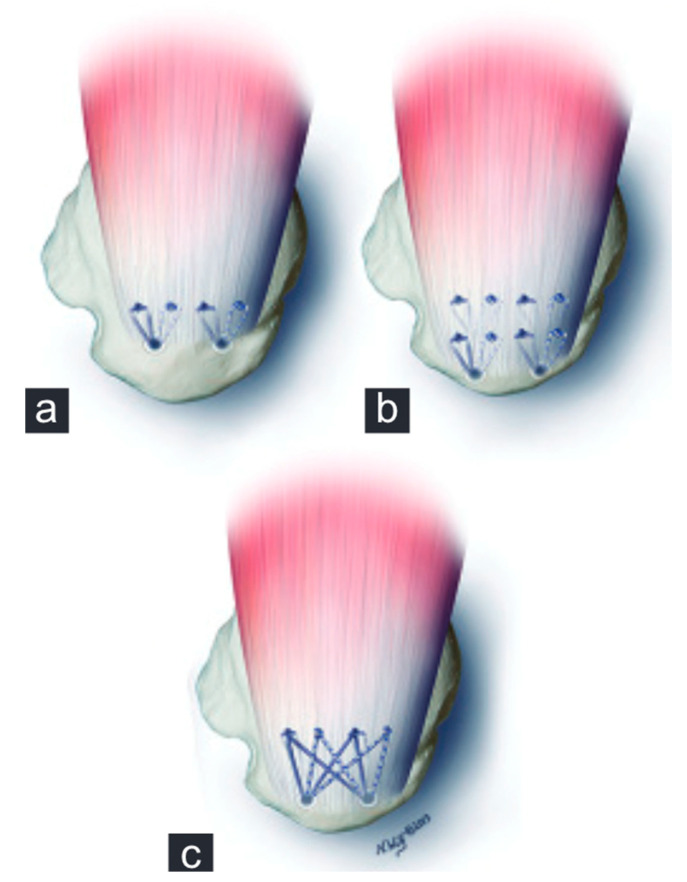
Illustration of (**a**) single row, (**b**) double row, and (**c**) suture bridge technique [84]. (Reproduced from with permission from Ref. [84]. Copyright © 2023, published by Medknow Publications. Free to read and use).

**Figure 8:**
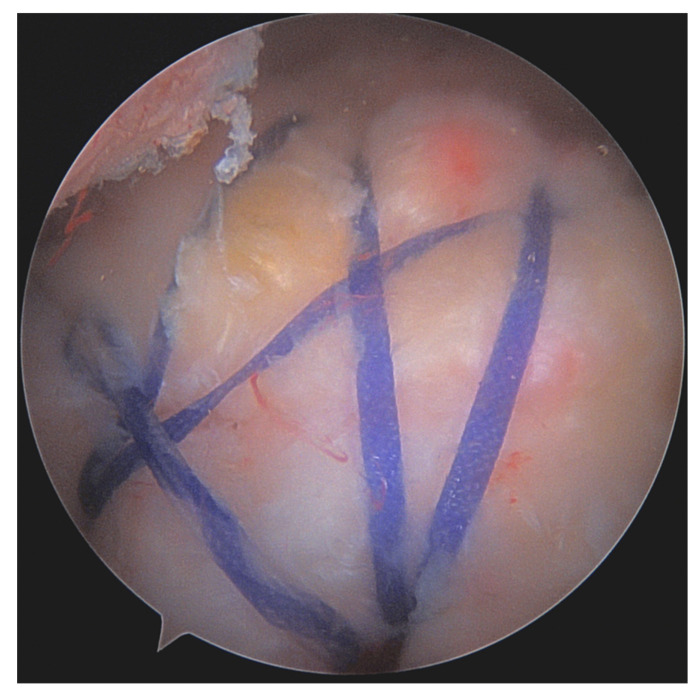
Arthroscopic image of a rotator cuff repair using the suture bridge technique.

With regard to fixation techniques, transosseous and single-row (SR) techniques have in recent years been largely replaced by double-row (DR) and transosseous-equivalent (TOE) or suture bridge techniques (Figure 7) using suture anchors. DR/suture bridge techniques were shown to be superior in multiple biomechanical studies in terms of anatomical coverage of the footprint, maximum load to failure under static and cyclic loading, and gapping between tendon and bone [78,79]. While in a classic DR technique loaded anchors are placed in the medial and lateral row, with sutures/tapes being shuttled and knotted independently, suture bridge techniques (Figure 8) only have loaded anchors placed in the medial row. The sutures/tapes are then shuttled through the tendon, typically in mattress configuration. This is best done medial of the rotator cable, as this is where they find the most stable anchorage [80]. Subacromially, either with or without prior medial knot tying, and in a crisscross configuration, the sutures are threaded into knotless anchors that are inserted into the lateral portion of the greater/lesser tuberosity—here best 15–21 mm distal to its tip for best purchase [81]. This configuration of anchor positions, stitches, and suture trajectory results in even load distribution and contact pressure of the tendon against the footprint [82]. By inserting the medially shuttled sutures/tapes in the lateral anchor, they lay smoothly on the tendon stump, avoiding subacromial knot impingement [83]. 

Interestingly, the biomechanical superiority of DR/suture bridge techniques over SR techniques has not yet been clearly reflected in clinical outcome studies. Several comparative analyses performed over the past decade have failed to demonstrate a difference in outcome scores [85,86]. Although healing rates and re-rupture rates have been shown to be in favor of DR/suture bridge techniques, for example, according to a 2022 meta-analysis by the Canadian Shoulder and Elbow Society [87] and a 2014 meta-analysis by Millet [88], respectively. Notably, a 2013 meta-analysis by Zhang et al. found that patients with large tears (>3 cm) and DR reconstruction had better functional outcomes than those with SR reconstruction [89]. There was, however, no difference in small tears. In 2022, Storti et al. found better flexion strength after DR/suture bridge in a comparative study of a total of 135 shoulders [90]. Thus, there seems to be a at least a tendency toward superior clinical results in DR reconstructions. 

### 4.3. Suture Anchor Systems and Materials

The goal of all suture anchor systems is to provide stable fixation of the reduced rotator cuff tendon to the greater/lesser tuberosity while biological healing occurs. This requires a strong purchase of the implant in the bone, secure fixation of the suture/suture tape to the anchor, and the best possible interconnection of suture/suture tape and tendon. Of course, the stability of the construct depends not only on the properties of the implant but also on the patient’s tissue and the choice and quality of the fixation construct. Osteoporotic bone or tendinopathic tendon tissue may entail failure, but so can surgical errors, such as malpositioning of sutures or anchors, or excessive removal of cortical bone at the footprint [80,81,91]. The most common mode of failure is suture cut-out through the tendon [92].

There is currently a wide variety of suture anchors available on the market. Materials include suture material only (“all-suture”), titanium, polyetheretherketone (PEEK), or bioresorbable materials (poly-L-lactide, poly-L,D-lactide +/− hydroxyapatite, (beta-)tricalcium phosphate). The latter have osteoconductive properties and become replaced by bone within about 2 years [93]. Like PEEK anchors, they allow artifact-free postoperative imaging. Artifacts are a disadvantage of titanium anchors, which otherwise offer excellent biomechanical properties and are therefore useful in certain situations, such as in revision surgeries or in osteopenic bone [94]. Solid anchors are available as screw-in or impaction anchors. All-suture anchors, which are, at this point, still relatively new, typically consist of one to two suture pairs made of UHMWPE (ultra-high-molecular-weight polyethylene) and polyester, which are connected to a more rigid tape/tube made of the same material. After insertion, this tape/tube unravels into a “ball” that anchors subcortically. All-suture anchors occupy the least volume within the bone. In terms of cyclic loading capacity and maximum failure load, they meet the required criteria but appear to allow slightly more micromovement and overall are still somewhat biomechanically inferior to the solid anchor types [95]. While their use in labral surgery is already established, their role in rotator cuff surgery is still being evaluated.

Not only the anchor’s shape and material, but also the type of suture is crucial. The usage of Ethibond (Ethicon, Somerville, NJ, USA), which was most widely employed for many years, has now been replaced by the usage of polyblend sutures made of UHMWPE and polyester, e.g., FiberWire (Arthrex, Naples, FL, USA). This material has been proven to be even more failure-resistant and is therefore now one of the most used materials. Other types of sutures combine the polyblend material with an absorbable polydioxanone (PDS) center. That way, high tensile strength is provided, but less suture material remains in the body [96]. Newer on the market are sutures with a central silicon-salt core, which expand in a moist environment and allow for higher and constant secondary stability (i.e., Dynacord, DePuy Mitek, Raynham, MA, USA). Even higher tensile strength than the mentioned sutures is achieved with suture tapes [97], which are in the meantime often used for rotator cuff reconstructions in knotless suture bridge techniques.

Today, a wide range of high-quality, technically innovative implants and materials are available to surgeons. However, whether their biomechanical properties allow for improved clinical outcome after rotator cuff repair remains to be seen and needs to be evaluated. 

### 4.4. Concomitant Acromioplasty

Acromioplasty as an adjunct to rotator cuff repair is still a routine surgical step in the hands of many surgeons. Assessment of the current literature delivers unequivocal results. Chen et al. [13] reported in a 2018 meta-analysis that adjunctive acromioplasty lead to significantly superior results with regard to shoulder pain relief at 12 months postoperatively and ASES score improvement at final follow-up. A randomized controlled trial by Woodmass et al. [98] with a follow-up of 11 years, on the other hand, concluded that there were no differences in the patient-reported clinical outcome; however, a significantly higher reoperation rate was observed in patients who had rotator cuff repair without acromioplasty. Conversely, a recent analysis of the Medicare database including over 50,000 shoulders and comparing the incidence of rotator-cuff-related revision surgery in patients with and without concurrent acromioplasty at index rotator cuff repair found a significantly increased rate of repeat cuff repair at 5 years postoperatively (8.5% vs. 6.8%, *p* < 0.001) in the acromioplasty group [99]. 

While these mentioned studies are well-conceived studies with regard to study design, sample size, and follow-up, they do not differentiate between different surgical techniques. Specifically, they do not discern “traditional” anterior acromioplasty geared toward elimination of the impinging spur [100] from lateral acromioplasty geared toward normalization of the CSA, thus hypothetically unloading the supraspinatus tendon, as proposed by Gerber [101]. Further research will likely deliver interesting results and guidance for future practice. 

### 4.5. Augmentation

**Figure 9:**
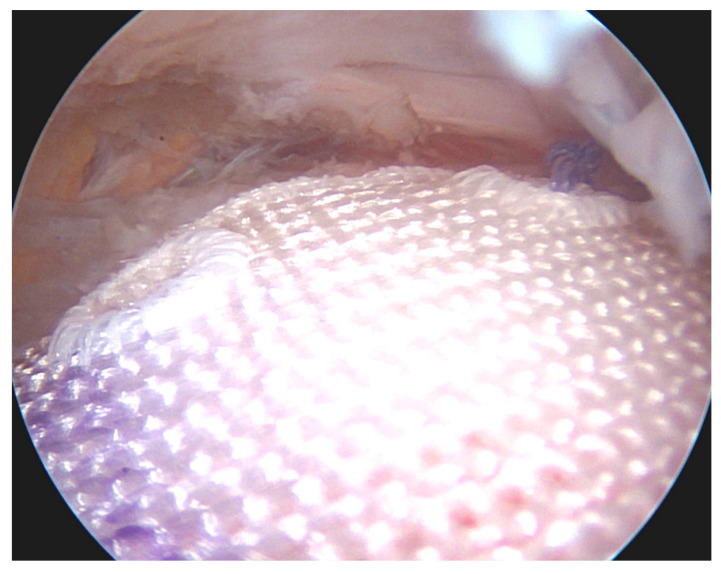
Arthroscopic rotator cuff repair using a synthetic patch (Pitch-Patch, Xiros Inc., Mansfield, MA, USA) for augmentation.

In cases of poor tendon quality, borderline reparability, or in the context of revision surgery, augmentation procedures may be considered. A variety of different materials are available, including allografts (mostly human acellular dermis), xenografts (e.g., porcine dermis, bovine pericardium), synthetic grafts (e.g., polyethylene terephthalate) (Figure 9), and various autografts (e.g., long biceps tendon) [51]. Various studies have demonstrated beneficial biomechanical properties of patch-augmented rotator cuff reconstructions, the majority of which were tested in vitro on cadaveric tissue [102,103,104,105,106,107]. To date, little is known about the in vivo response of tendon tissue augmentation. In a 2020 biomechanical cadaveric study by Mehta et al. that examined an absorbable BioFiber patch of poly-4-hydroxybutyrate, they found reduced gapping and higher load to failure under cyclic loading compared to patch-free reconstruction [108]. As to the in vivo response of tendon tissue, Rashid et al. performed early postoperative biopsies in three groups of patients who had undergone different types of RC repair: (1) augmented with acellular human dermis, (2) augmented with acellular cross-linked porcine dermis, and (3) control group without patch. Histologically, the study revealed significant disruptions of the extracellular tendon matrix adjacent to the grafts [109]. Good clinical and radiological follow-up studies are also still scarce. In 2022, a prospective randomized case–control study by Lee et al. [110] demonstrated promising results with medium-term follow-up of approximately 5.7 years. Patients with massive tears who received additional allograft augmentation (acellular human dermis) had a significantly lower retear rate than those without patch (9.1% vs. 38.1%, *p* = 0.034). Furthermore, subgroup analysis showed better results in both groups when complete footprint coverage was achieved intraoperatively [39]. Clinical scores (Constant score (CS), American Shoulder and Elbow Score (ASES)) improved in both groups, with the degree of improvement being significantly higher in the patch group than in the nonpatch group. 

Becoming increasingly popular is the use of autografts and biologically active substances, for instance, by use of the long head of biceps tendon. Different techniques have been described, for example, by Colbath et al. [111], who presented a technique, where, by producing a mesh graft, similar to skin mesh graft, a scaffold is created. While the graft’s biomechanical properties (ultimate tensile force, tensile modulus) are inferior to those of a native rotator cuff tendon, it theoretically contained viable tenocytes, thus allowing for biological augmentation of the repaired rotator cuff. As to outcomes after biceps autograft augmentation of rotator cuff repairs, a 2018 review article summarizing eight case series including a total of 170 patients concluded that results were comparable to other types of augmentation. One hundred and twelve patients received a follow-up MRI; sixty-eight of which had intact reconstructed tendons [112]. 

Klatte-Schulz et al. recently authored an article in which they describe the potential clinical use of the subacromial bursa as a biologic augment of the rotator cuff. Both its tight fibrovascular structure and its high content of growth factors and progenitor cells were highlighted [113]. In the same context, Freislederer et al. have published a technique using the parietal sheet of the subacromial bursa to augment their rotator cuff repair at the level of the footprint [114], whereas Bhatia has described a technique utilizing the bursa to biologically augment a long biceps tendon autograft [115]. Further clinical studies on combined mechanical and biological augmentation have been published by Muench [116,117], Berthold [118], and Wellington [119]. In summary, they report on combinations of dermal allografts with subacromial bursa, platelet-rich and platelet-poor plasma (PRP, PPP), concentrated bone marrow aspirate, and autologous thrombin. However, clinical outcomes using these augments have not yet been reported to date. 

Overall, the role of rotator cuff augmentation is still unclear. To date, the various products available on the market have been used very heterogeneously [120], which renders the interpretation of previously published literature difficult. Naturally, the added cost needs to be taken into consideration. Interestingly, a research group based in the United Kingdom is currently conducting a large-scale randomized controlled trial on the subject. The results and conclusions of the Patch Augmented Rotator Cuff Surgery (PARCS) project are highly anticipated and may alter future management [121,122]. 

## 5. Postoperative Rehabilitation

The necessity, nature and duration, of postoperative immobilization following rotator cuff surgery is still a topic of debate. However, in recent years, it is safe to say that the traditional practice of postoperative immobilization in abduction has been challenged in several studies [123,124,125,126].

### 5.1. Immobilization Versus Nonimmobilization

In 2019, Tirefort et al. published a randomized control trial (level I), in which a total of 80 consecutive ARCR (DR technique) of small- to medium-sized (<3 cm) isolated superior rotator cuff lesions were included. Patients were randomized into two treatment arms: (1) no sling and (2) sling for 4 weeks. The nonimmobilized group presented improved early mobility and better functional scores without being affected by more retears (evaluated sonographically 6 months postoperatively) [123]. A larger cohort (206 patients) with a follow-up of 24 months was studied by a Canadian group led by Sheps et al. [124]. The patients in one study arm received a sling and were allowed passive motion only for 6 weeks. The patients in the other treatment arm were allowed to remove the sling as needed and to actively mobilize under pain-free conditions. Twelve months postoperatively, no significant differences were found between groups, neither clinically nor sonographically. These very well-designed studies have generated a wide response in the scientific community [120,123]. Along these lines, a 2021 systematic review on early versus delayed mobilization by Longo et al., which included a total of 16 level I and II studies with a total of 1424 patients, showed that early mobilization offered slight advantages in terms of mobility in the early postoperative period. However, no difference was observed after 24 months, neither in terms of mobility, nor functional scores, nor retear rate (9.5% vs. 11.4%, *p* = 0.29) [127]. In summary, according to these studies published between 2019 and 2021, immobilization may be avoided, and cautious early active motion may be permitted and advisable, at least in patients after reconstruction of small- to medium-sized tears. 

In contrast to these results, however, are the findings of Grubhofer et al. They conducted a two-part examination, where firstly, the brace wearing compliance of 50 patients was tested by means of a sensor integrated into an abduction brace; and then secondly, they reported the patients’ clinical and structural follow-up data, the latter being assessed sonographically, and if applicable, using MRI. Their results suggested that patients with low compliance have higher retear rates, and that patients with retears perform comparatively worse in terms of clinical parameters [128,129]. Given these results, it is recommended that a conservative approach be taken concerning the postoperative management of our rotator cuff patients, with relative immobilization for 6 weeks postoperatively, allowing passive movement exercises within certain limits. 

### 5.2. Sling Versus Abduction Brace

The intention of immobilizing the shoulder in abduction after rotator cuff surgery is to reduce tension on the healing supra-/infraspinatus tendon and thus to improve conditions for healing. The abduction brace can be slightly complicated to apply and, for many, uncomfortable to wear. Furthermore, its benefits to patient outcomes are currently being challenged, although the literature certainly supports the concept. Schenk et al., for example, found that load transfer to the brace is biomechanically most favorable at 30–50° of abduction in the scapular plane [130]. Interestingly, Pandey et al. who sonographically assessed blood flow at the site of the reconstructed posterosuperior rotator cuff in 30° abduction and in the 0° position, both immediately and at 6 weeks postoperatively, found increased blood flow in the abducted position. Despite this presumably being an advantage for tendon healing, no clinical superiority could be demonstrated when measured by the visual analogue scale (VAS) for pain and the Constant score. There were also no differences as to tendon integrity on sonogram [131]. Hollman et al. [125] and Ghandour et al. [126] also presented equivalent results in terms of clinical outcomes in prospective randomized trials. The latter group included the measurement of isokinetic external rotation force at the 1-year follow-up as an additional outcome parameter and again found no significant difference between the two groups. 

In summary, clear evidence supporting the use of an abduction brace is lacking. Nevertheless, based on its theoretical advantages, in patients with medium- to large-sized (postero)superior rotator cuff reconstructions, this continues to be our preference. On the other hand, in patients with small or partial reconstructions or in those who are not able to tolerate an abduction brace, a conventional broad arm sling is used.

## 6. Future Directions

The first documented open rotator cuff repair was performed in 1870 by Karl Hüter. In the early 20th century, repairs were thought to be particularly strong when “the sutures were knotted distal to the tip of the greater tuberosity over a wide bridge of bone and cortical anchorage occurred” [132,133]. In 1962, McLaughlin stated that successful rotator cuff reconstruction required tightly adherent, low-tension, continuity-restoring positioning of healthy tendon tissue, with watertight closure on a smooth surface [134]. Over the last 100 years, there have been significant advances in understanding, techniques, and implant technology. Nevertheless, the treatment principles still remain valid. Today’s arthroscopic rotator cuff repair techniques and modern implants facilitate reliable fixation to allow for tendon–bone healing. New developments focus on methods and devices that aim to provide the best possible mechanical properties, to spare bone and biologically active tissues, and to augment and protect the repairs. At the same time, efforts are being undertaken to help clinicians and patients conceptualize individually tailored therapeutic approaches using today’s sophisticated technology on the one hand, but also taking into consideration patients’ genetic predispositions, lifestyle, as well environmental factors, the objective being to obtain more reliable and efficient results [135]. Current key points and recommendations regarding the treatment of rotator cuff tears are provided in Table 2. It is anticipated that future research will be focused on exploring deeper into the pathology itself to help refine patient selection and treatment indication. 

## Figures and Tables

**Table 1:** Prognostic factors for good outcome after rotator cuff reconstruction.

Age <65 years
Small anteroposterior tear size
Little tendon retraction
No/minor fatty infiltration Long tendon stump (>15 mm)
Early intervention for traumatic tears

**Table 2:** Key points and recommendations for treatment of rotator cuff disorders.

Imaging	Gold Standard: MRI
	- oblique axial, sagittal, and coronal planes - T1-weighted and fluid-sensitive sequences - consider additional sequences, MR arthrography or ultrasound for specific questions
Treatment Indication	Degenerative tears deteriorate slowly and can undergo a conservative treatment trial
	- Cave: involvement of the rotator cable - Cave: large tears - Cave: incipient fatty muscle infiltration
	Traumatic rotator cuff tears should be addressed surgically soon after trauma.
Fixation Technique	DR/suture bridge techniques are biomechanically superior to SR/transosseus techniques.
	Modern anchor and suture materials facilitate stable fixation.
	Consider mechanical and/or biological augmentation in - poor tendon quality - borderline reparability - revision surgery
Postoperative Rehabilitation	Small/partial rotator cuff repairs: conventional broad arm sling
	Medium-/large rotator cuff repairs (incl. infraspinatus): abduction brace
